# Disease Activity Trajectories During Pregnancy Predict Medically Indicated Complications in Women with Systemic Lupus Erythematosus

**DOI:** 10.3390/jcm15072774

**Published:** 2026-04-07

**Authors:** Meng Jiang, Yanling Chang, Wen Di, Jiayue Wu

**Affiliations:** 1 Department of Obstetrics and Gynecology, Renji Hospital, School of Medicine, Shanghai Jiao Tong University, Shanghai 200127, China; jiangmeng016369@renji.com (M.J.); changyanling@renji.com (Y.C.); 2 Shanghai Key Laboratory of Gynecologic Oncology, Shanghai 200127, China; 3 State Key Laboratory of Oncogenes and Related Genes, Shanghai Cancer Institute, Renji Hospital, School of Medicine, Shanghai Jiao Tong University, Shanghai 200127, China

**Keywords:** systemic lupus erythematosus, pregnancy, disease activity trajectory, uncontrolled disease activity, pregnancy complications

## Abstract

**Background**: Pregnancy in women with systemic lupus erythematosus (SLE) carries an increased risk of maternal and obstetric complications. Current risk assessment often relies on disease activity measured at a single time point and may not reflect dynamic changes during pregnancy. The clinical value of longitudinal disease activity trajectories remains insufficiently defined. **Methods**: We conducted a retrospective cohort study of 245 pregnancies in women with SLE managed at a tertiary referral center. Disease activity was assessed longitudinally and classified into three trajectories: persistent low activity, early flare with subsequent control, and uncontrolled disease activity during pregnancy. The primary outcome was a composite of clinically actionable maternal or obstetric complications prompting medical intervention. Multivariable logistic regression was performed to evaluate associations between disease activity trajectories and the primary outcome. **Results**: Clinically actionable pregnancy complications occurred in 41 out of 245 pregnancies (16.7%). The incidence differed significantly across disease activity trajectories, occurring in 3.9% of pregnancies with persistent low activity, 11.6% with early flare followed by control, and 63.8% with uncontrolled disease activity during pregnancy (*p* < 0.001). After adjustment for relevant covariates, uncontrolled disease activity remained strongly associated with the primary outcome (adjusted odds ratio 13.45, 95% confidence interval 4.01–45.08), whereas early flare with subsequent control was not associated with increased risk. **Conclusions**: Disease activity trajectories during pregnancy are strongly associated with clinically actionable pregnancy complications in women with SLE. Uncontrolled disease activity confers a markedly increased risk, while early disease flare followed by effective control does not. Trajectory-based assessment may improve risk stratification and support more individualized management during SLE pregnancies.

## 1. Introduction

Systemic lupus erythematosus (SLE) predominantly affects women of reproductive age and poses substantial challenges during pregnancy. Despite advances in multidisciplinary care, pregnant women with SLE continue to experience higher rates of maternal and fetal complications compared with the general obstetric population, including preeclampsia, preterm birth, and medically indicated delivery [1,2]. Accurate risk stratification remains central to optimizing pregnancy management in this high-risk population.

Previous studies have consistently identified several predictors of adverse pregnancy outcomes in women with SLE, such as active disease at conception, lupus nephritis, antiphospholipid antibodies, and immunological abnormalities, and a history of adverse pregnancy outcomes in prior pregnancies [3,4]. Most existing risk assessment models rely on static evaluations of disease activity, typically measured at a single time point before or early in pregnancy, most commonly using baseline SLEDAI scores or dichotomized activity status (active versus inactive) [5,6,7].

However, pregnancy represents a dynamic immunological and physiological state, during which disease activity in SLE may fluctuate substantially. A single static assessment may fail to capture the longitudinal evolution of disease activity throughout gestation. Importantly, whether distinct patterns of disease activity over time, rather than baseline activity alone, convey incremental prognostic information for clinically meaningful pregnancy outcomes remains largely unexplored.

Emerging evidence in other chronic inflammatory and autoimmune conditions suggests that disease trajectories, reflecting the direction and stability of disease activity over time, may be more informative than isolated measurements in predicting adverse outcomes [8,9]. In the context of SLE pregnancy, longitudinal patterns of disease activity—such as disease flares occurring early in pregnancy [10], sustained low disease activity [11], or recurrent fluctuations—may capture clinically relevant differences in risk profiles and disease courses, beyond what can be inferred from isolated assessments at single time points [12]. Yet, data addressing this trajectory-based approach in pregnant women with SLE are scarce.

Moreover, many prior investigations have relied on composite adverse pregnancy outcomes, which—while informative for epidemiologic description—may obscure the specific, decision-relevant events that prompt obstetric intervention. In women with SLE a substantial proportion of preterm births are medically indicated rather than spontaneous [13,14], and these indicated deliveries often reflect acute clinical inflection points (for example, severe preeclampsia, worsening maternal organ dysfunction, or progressive fetal compromise) that directly alter management [15]. Focusing on such clinically actionable endpoints (e.g., medically indicated preterm birth and severe maternal complications prompting delivery) therefore better aligns research with the realities of multidisciplinary decision-making and may provide more immediately translatable information for clinicians caring for pregnant women with SLE [16].

Therefore, this study aimed to investigate whether distinct disease activity trajectories during pregnancy are associated with clinically relevant maternal and fetal outcomes in women with SLE. Using a retrospective cohort from a tertiary referral center, we categorized pregnant women with SLE based on longitudinal patterns of disease activity and examined their associations with outcomes that directly influence clinical decision-making, with a focus on clinically actionable maternal or obstetric complications prompting intervention, such as medically indicated preterm birth and severe maternal morbidity [16]. This trajectory-based approach may provide novel insight beyond static measures of disease activity and better inform individualized risk stratification and management strategies in this high-risk population.

## 2. Materials and Methods

### 2.1. Study Design and Population

This retrospective cohort study was performed at Ren Ji Hospital, a tertiary referral center specializing in high-risk obstetric care. Pregnant women with a confirmed diagnosis of SLE, established according to the 2012 Systemic Lupus International Collaborating Clinics (SLICC) criteria, were consecutively identified from the institutional obstetric database between January 2020 and December 2024.

Eligible participants included women with singleton pregnancies who were followed throughout gestation and had available clinical assessments of disease activity. Pregnancies complicated by other systemic autoimmune diseases were excluded. Women with antiphospholipid syndrome as the predominant indication for pregnancy management were not excluded but were adjusted for in multivariable analyses.

This retrospective cohort study protocol was initially approved by the Institutional Ethics Committee of Renji Hospital on 25 December 2019 (approval number: KY2019-138), renewed on 20 February 2022 (approval number: KY2021-199-B), and further renewed on 28 September 2025 with the same approval number KY2021-199-B.

### 2.2. Assessment of Disease Activity

Disease activity was assessed using the Systemic Lupus Erythematosus Disease Activity Index (SLEDAI) when formally recorded, supplemented by detailed clinical documentation when complete SLEDAI scores were unavailable. Disease activity assessments were extracted at three predefined windows: preconception or early pregnancy (first trimester), mid-pregnancy (second trimester), and late pregnancy, when applicable. Accordingly, whenever possible, disease-activity information used for trajectory classification was obtained before the outcome-defining intervention or event.

Active disease was defined according to established clinical criteria, including new or worsening organ involvement, immunological abnormalities, or escalation of immunosuppressive therapy. When available, serologic activity (e.g., complement consumption and/or anti-dsDNA changes) was captured through formal SLEDAI scoring; otherwise, it was abstracted from contemporaneous clinical documentation used for trajectory assignment. When formal SLEDAI scores were unavailable, disease activity was adjudicated based on contemporaneous clinical documentation by treating rheumatologists, consistent with routine clinical practice.

### 2.3. Definition of Disease Activity Trajectories

To characterize longitudinal patterns of disease activity during pregnancy, participants were classified into three primary disease activity trajectory groups based on clinical assessments across predefined windows. Trajectories were defined *a priori* by expert consensus to reflect clinically interpretable patterns of disease evolution rather than data-driven statistical clustering, and their reproducibility in external cohorts remains to be established.

The following trajectory categories were used for the primary analyses:

- Persistent low activity: sustained low or inactive disease throughout pregnancy without a clinician-documented flare requiring treatment escalation (e.g., increase in systemic glucocorticoids and/or initiation/escalation of immunosuppressive therapy) and without new or worsening organ involvement attributable to SLE.
- Early flare with subsequent control: disease activity present before or during early pregnancy that improved and remained controlled after treatment adjustment;
- Uncontrolled disease activity during pregnancy: disease activity that was not stably controlled during pregnancy, including either new-onset disease activity after conception or progression of pre-existing activity.

Trajectory assignment was performed independently by two clinicians, blinded to pregnancy outcomes. Disagreements were adjudicated in a consensus meeting; if disagreement persisted, a third senior clinician made the final determination. Inter-rater agreement was quantified using Cohen’s kappa. The number and proportion of discordant cases are reported in the Section 3.

In secondary and descriptive analyses, the uncontrolled disease activity was further subdivided into:

1. de novo disease activity during pregnancy, defined as the first occurrence of clinically significant SLE activity after conception in women without evidence of active disease prior to or in early pregnancy; and
2. progressive or worsening disease activity, defined as persistence or escalation of disease activity during pregnancy despite pre-existing activity.

### 2.4. Outcomes

#### 2.4.1. Primary Outcome

The primary outcome was a composite of clinically significant maternal or obstetric complications that directly prompted obstetric intervention, defined as the occurrence of one or more of the following:

- medically indicated preterm birth (<37 weeks of gestation);
- severe preeclampsia requiring delivery;
- maternal organ dysfunction leading to pregnancy termination.

Component outcomes were not mutually exclusive; therefore, component counts may exceed the composite total. This composite endpoint was selected to capture management-altering events with a shared clinical consequence (obstetric intervention), thereby improving clinical interpretability and statistical efficiency in a cohort with a limited number of events.

For each pregnancy, the timing of the primary outcome was anchored to the first clinically actionable event prompting intervention, typically the date of delivery or pregnancy termination. When an outcome occurred before a scheduled late-pregnancy assessment, trajectory classification relied on the last available disease-activity assessment prior to the event.

#### 2.4.2. Secondary Outcomes

Secondary outcomes included spontaneous preterm birth, fetal growth restriction, mode of delivery, and primary indication for cesarean section, as well as neonatal outcomes including birth weight, Apgar scores, and neonatal intensive care unit admission.

### 2.5. Covariates

Baseline and pregnancy-related covariates included maternal age, parity, history of prior adverse pregnancy outcomes, disease duration, history of lupus nephritis, history of neuropsychiatric SLE (NP-SLE) manifestations, antiphospholipid antibody status, medication use (including hydroxychloroquine and glucocorticoids), and relevant laboratory parameters. aPL positivity was defined as a positive result for any antiphospholipid antibody test (lupus anticoagulant, anticardiolipin, and/or anti-β2-glycoprotein I) recorded in the medical record. Detailed aPL profile stratification (including single-, double-, or triple-positivity and antibody titers) was not sufficiently complete and standardized across the retrospective record to support reliable subgroup analysis. Systemic glucocorticoid exposure was coded as a prednisone-equivalent daily dose >10 mg/day during pregnancy, based on medication records. These variables were selected *a priori* based on clinical relevance and prior literature.

### 2.6. Statistical Analysis

Continuous variables were summarized as the mean ± standard deviation or median with interquartile range, as appropriate, and compared using analysis of variance or the Kruskal–Wallis test. Categorical variables were compared using the chi-square test or Fisher’s exact test, with Monte Carlo exact methods applied when sparse cell counts precluded reliable asymptotic assumptions.

Multivariable logistic regression models were constructed to evaluate the association between disease activity trajectories and the primary outcome, adjusting for clinically relevant covariates.

For the primary analyses, disease activity trajectories were modeled as three categories. Secondary and sensitivity analyses were performed to descriptively examine outcomes within subtypes of uncontrolled disease activity.

Results were reported as odds ratios (ORs) with 95% confidence intervals (CIs). Collinearity among covariates was assessed prior to model construction.

Sensitivity analyses were conducted to evaluate the robustness of the primary findings. To reduce potential misclassification arising from incomplete formal SLEDAI documentation, we repeated the primary multivariable model in the subset of pregnancies with formally recorded SLEDAI scores available across all three assessment windows. Missing data were handled using a complete-case approach for the primary multivariable model (pregnancies with missing values in any model covariate were excluded from that analysis). No observations were excluded due to missing covariate data. Model fit was evaluated using the Hosmer–Lemeshow goodness-of-fit test, and discriminatory performance was quantified by the area under the receiver operating characteristic curve (AUC/C-statistic) based on the model-predicted probabilities. No stepwise selection procedures were used. A two-sided *p* value <0.05 was considered statistically significant. All analyses were conducted using SPSS version 29.0.

## 3. Results

### 3.1. Study Population and Disease Activity Trajectories

A total of 245 pregnancies in women with systemic lupus erythematosus were included in the analysis, and all were followed through delivery. Based on longitudinal assessments of disease activity during pregnancy, participants were classified into three disease activity trajectory groups: persistent low activity (*n* = 155), early flare followed by disease control (*n* = 43), and uncontrolled disease activity during pregnancy (*n* = 47). Formal SLEDAI scores across all three assessment windows were available in 198 out of 245 pregnancies (80.8%), whereas 47 pregnancies (19.2%) had incomplete formal SLEDAI documentation. Inter-rater agreement for trajectory assignment was excellent (Cohen’s κ = 0.962, 95% CI 0.929–0.995, *p* < 0.001). Discordant classifications occurred in 5/245 (2.0%) pregnancies and were resolved by consensus; all disagreements were between the persistent low and early flare with subsequent control categories; no discordance involved the uncontrolled category. The distribution of disease activity trajectories is presented in Table 1.

### 3.2. Baseline Characteristics Across Disease Activity Trajectories

Baseline clinical characteristics according to disease activity trajectories are summarized in Table 1. Mean maternal age was similar across the three trajectory groups. A history of lupus nephritis differed significantly by trajectory and was more common among women with uncontrolled disease activity than among those with persistent low activity. Antiphospholipid antibody positivity and steroid dose distribution also differed across trajectory groups, whereas hydroxychloroquine use was comparable.

### 3.3. Incidence of Clinically Actionable Pregnancy Complications

Overall, the primary outcome occurred in 41 out of 245 pregnancies (16.7%). Among pregnancies meeting the composite endpoint, medically indicated preterm birth occurred in 38 cases, severe preeclampsia requiring delivery in 31 cases, and maternal organ dysfunction leading to pregnancy termination in 16 cases. These component outcomes were not mutually exclusive. The incidence differed markedly across disease activity trajectories. Clinically actionable complications occurred in 6 out of 155 pregnancies (3.9%) among women with persistent low activity, 5 out of 43 pregnancies (11.6%) among those with early flare followed by disease control, and 30 out of 47 pregnancies (63.8%) among women with uncontrolled disease activity during pregnancy (*p* < 0.001, Table 2).

### 3.4. Multivariable Analysis

**Figure 1:**
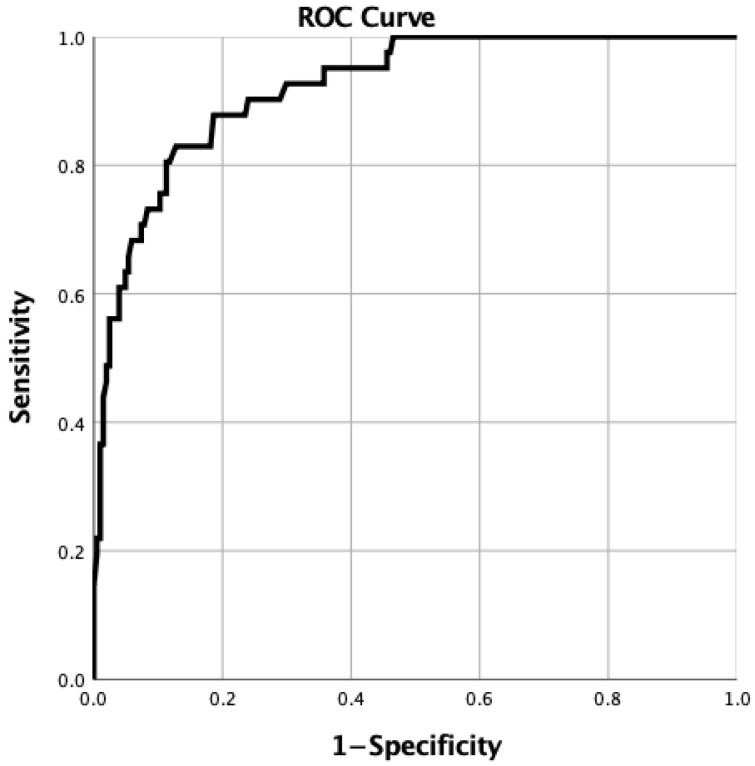
Receiver operating characteristic (ROC) curve for the primary multivariable logistic regression model. The ROC curve was constructed using model-predicted probabilities for clinically actionable pregnancy complications. The model showed excellent discrimination (AUC = 0.921).

Multivariable logistic regression analyses were performed with adjustment for maternal age, history of lupus nephritis, antiphospholipid antibody status, hydroxychloroquine use, and steroid dose. All 245 pregnancies had complete covariate data and were included in the multivariable model. The primary model included 41 outcome events and 7 regression parameters, corresponding to approximately 5.9 events per parameter. Disease activity trajectories remained independently associated with the primary outcome (overall *p* < 0.001). Compared with women with persistent low activity, those with uncontrolled disease activity during pregnancy had markedly higher odds of clinically actionable complications (adjusted odds ratio 13.45, 95% confidence interval 4.01–45.08, *p* < 0.001). In contrast, women with early flare followed by subsequent control did not show a significant difference (adjusted odds ratio 1.03, 95% confidence interval 0.24–4.49, *p* = 0.97). Given the limited number of primary outcome events (41 events) relative to the number of model parameters, the estimated effect sizes—particularly for the uncontrolled trajectory—should be interpreted cautiously, as reflected by the wide confidence intervals. Accordingly, the point estimates should be interpreted as evidence of a strong association rather than a precise quantification of risk. Prednisone-equivalent dose >10 mg/day also showed an association with the primary outcome; however, this finding should be interpreted as a marker of more severe or unstable disease and/or treatment escalation rather than as evidence of a direct causal glucocorticoid effect. The adjusted model showed acceptable fit (Hosmer–Lemeshow χ^2^ = 4.008, df = 8; *p* = 0.856) and excellent discrimination (AUC = 0.921, 95% CI 0.880–0.962, *p* < 0.001) (Figure 1). Full model results are shown in Table 3.

### 3.5. Subgroup Analysis Within Uncontrolled Disease Activity Trajectories

Within the uncontrolled disease activity group, 10 women developed de novo disease activity during pregnancy, while 37 experienced progressive disease activity. Clinically actionable complications occurred in 7 out of 10 women (70.0%) with de novo disease activity and in 23 out of 37 women (62.2%) with progressive disease activity. Given the limited sample size of these subgroups, formal comparative statistical testing was not performed.

### 3.6. Sensitivity Analyses

In sensitivity analyses, the association between uncontrolled disease activity during pregnancy and clinically actionable complications remained robust after adjustment for the same covariates. Specifically, in the complete-SLEDAI subset (*n* = 198), disease-activity trajectory remained independently associated with clinically actionable pregnancy complications after adjustment for the same covariates (overall *p* < 0.001). Baseline characteristics and outcome distribution were broadly similar between pregnancies with complete and incomplete formal SLEDAI documentation. Compared with the persistent-low trajectory, the early flare with subsequent control trajectory was not associated with higher odds of complications (adjusted OR 0.89, 95% CI 0.14–5.62; *p* = 0.897), whereas uncontrolled disease activity remained strongly associated with complications (adjusted OR 20.05, 95% CI 4.46–90.19; *p* < 0.001). History of lupus nephritis (adjusted OR 2.15, 95% CI 1.20–3.83; *p* = 0.010) and steroid dose >10 mg/day (adjusted OR 4.66, 95% CI 1.03–21.08; *p* = 0.046) also remained significant. These findings were consistent with the primary analysis (Table 3).

### 3.7. Secondary Pregnancy Outcomes

Spontaneous preterm birth occurred in 43 out of 245 pregnancies (17.6%) and did not differ significantly across disease activity trajectory groups (*p* = 0.53). In contrast, medically indicated complications accounted for the majority of adverse outcomes among women with uncontrolled disease activity. Detailed results are shown in Table 2.

## 4. Discussion

In pregnancies complicated by systemic lupus erythematosus, disease activity has long been recognized as a key determinant of adverse maternal and fetal outcomes. Previous observational studies have shown that higher disease activity during pregnancy is associated with increased risk of adverse pregnancy outcomes, including preterm delivery and other complications [17]. In addition, disease activity at conception or during early pregnancy has been shown to predict subsequent pregnancy risk [18]. However, most prior work has relied on baseline disease status or isolated measures of disease activity, rather than capturing longitudinal disease activity trajectories over time. In the present study, we extend these observations by classifying longitudinal disease activity into distinct trajectory groups, and we demonstrate that women with uncontrolled disease activity have a substantially higher incidence of clinically actionable pregnancy complications than those with persistent low activity or early disease flare that is subsequently controlled. These findings suggest that longitudinal disease activity trajectories, rather than single time-point assessments, may better identify high-risk pregnancies in women with SLE.

From a clinical perspective, our findings have several important implications for the management of pregnancy in women with systemic lupus erythematosus. Current clinical practice emphasizes disease quiescence at conception as a key prerequisite for favorable pregnancy outcomes, a principle supported by international recommendations. While this remains essential, our results suggest that risk assessment should extend beyond baseline status and incorporate continuous evaluation of disease activity throughout pregnancy. Women who experienced early disease flare but achieved sustained control did not demonstrate a significantly increased risk, highlighting the potential benefit of timely intervention and effective disease control during gestation, consistent with guideline-based management strategies [19].

Second, the identification of uncontrolled disease activity trajectory as a high-risk trajectory underscores the need for closer surveillance and proactive, individualized management in this subgroup. International guidelines for SLE pregnancy management consistently recommend frequent disease monitoring, multidisciplinary care, and prompt treatment adjustment in patients with active or unstable disease during pregnancy [19,20]. Our findings provide empirical support for these recommendations by demonstrating that fluctuating or progressive disease activity over time is associated with a markedly higher burden of clinically actionable pregnancy complications. Together, these results support a dynamic approach to risk stratification, in which longitudinal disease activity trajectories inform clinical decision-making more effectively than static, single time-point assessments.

Importantly, our findings provide quantitative evidence that complements existing recommendations by illustrating the magnitude of risk associated with different disease-activity trajectories during pregnancy. In our cohort, clinically actionable pregnancy complications occurred in nearly two-thirds of women with uncontrolled disease activity compared with fewer than one in ten women with persistent low activity. Even after adjustment for established risk factors, uncontrolled disease activity remained associated with a more than tenfold increase in the odds of complications. In contrast, women who experienced early disease flare but achieved sustained disease control showed outcomes comparable to those with persistently low activity.

Medically indicated preterm birth was the most frequent component of the composite endpoint, underscoring the management-altering nature of the outcome. Component outcomes were not mutually exclusive and could co-occur.

In addition to disease activity trajectories, prednisone-equivalent dose > 10 mg/day was associated with clinically actionable complications. Because glucocorticoid dose is frequently increased in response to flares and organ-threatening activity, this association is vulnerable to confounding by indication and is best viewed as a risk marker rather than evidence that glucocorticoids directly cause specific obstetric complications. Notably, high-dose steroid exposure was common in the ‘early flare with subsequent control’ group, yet this group did not experience excess risk compared with persistent low activity. This pattern supports the interpretation that achieving and maintaining disease control—rather than glucocorticoid dose alone—may be the dominant determinant of clinically actionable complications. From a practical standpoint, the need for >10 mg/day during pregnancy may serve as a bedside “red flag” warranting closer maternal–fetal surveillance and timely multidisciplinary optimization of therapy to achieve sustained disease control while minimizing prolonged high-dose exposure.

These data suggest that not all disease activity during pregnancy carries the same prognostic significance, and that the ability to achieve and maintain disease control may be a critical modifier of pregnancy risk. From a clinical standpoint, this trajectory-based framework may help clinicians move beyond binary classifications of “active” versus “inactive” disease and support more nuanced counseling and monitoring strategies. In this context, our study bridges guideline-level recommendations with real-world outcome data, offering an empirically grounded approach to identifying pregnancies at highest risk and those in whom favorable outcomes remain achievable with effective disease control.

Several limitations of this study should be acknowledged. First, the retrospective design may introduce inherent selection bias, and causal relationships cannot be definitively established. Because disease activity was assessed in trimester-based windows and outcomes occurred during pregnancy, partial temporal overlap—particularly in late pregnancy—cannot be fully excluded; thus, the observed associations should not be interpreted as establishing a strict causal sequence. Glucocorticoid dose may reflect treatment escalation for active or organ-threatening disease (confounding by indication) and may also contribute to obstetric risk; therefore, residual confounding cannot be ruled out. In addition, the number of outcome events was modest relative to the number of adjusted parameters, and the regression estimates—particularly for the uncontrolled trajectory—may be imprecise and potentially unstable, as reflected by the wide confidence intervals. Although longitudinal disease activity data were systematically collected, unmeasured confounding factors may still have influenced the observed associations. We analyzed aPL status as a binary variable (any aPL positive) without stratification by lupus anticoagulant, antibody titers, or single/double/triple positivity, and heterogeneity across aPL profiles may have obscured differential risks. Complement levels (C3/C4) were not uniformly available across assessment windows and were not included as separate covariates. Second, this single-center study from a tertiary referral institution likely reflects a higher-risk population than unselected SLE pregnancies, which may limit external validity and inflate absolute event rates relative to lower-acuity settings. External validation in multicenter cohorts is therefore warranted before broader application. Third, although the overall cohort size was moderate, some trajectory subgroups—particularly de novo activity during pregnancy—were small; subgroup analyses were therefore descriptive, and formal comparisons were not performed. Fourth, trajectories were derived from routinely collected clinical assessments, which may not fully capture short-term fluctuations between visits. Given the sparse, window-based assessments and incomplete formal SLEDAI recording, we prioritized interpretability and reproducibility by using prespecified, clinically defined trajectories; under these constraints, data-driven latent trajectory modeling would likely be unstable, and our categories should be interpreted as pragmatic clinical patterns rather than latent biological classes.

Finally, information on certain potential modifiers, such as cumulative disease duration prior to pregnancy and detailed treatment changes over time, was limited. Future prospective, multicenter studies with larger sample sizes and more granular longitudinal data are warranted to validate our findings and further clarify the clinical utility of trajectory-based risk stratification in SLE pregnancies.

## 5. Conclusions

In this retrospective cohort of pregnancies complicated by SLE, disease activity trajectories during pregnancy were strongly associated with clinically actionable pregnancy complications. Uncontrolled disease activity was linked to a markedly higher risk, whereas early disease flare followed by sustained control was not associated with adverse outcomes. By capturing dynamic patterns of disease activity rather than relying on single time-point assessments, this trajectory-based approach provides clinically relevant insights for risk stratification during pregnancy. These findings support the value of longitudinal disease monitoring and may inform more individualized management strategies in pregnant women with SLE.

## Figures and Tables

**Table 1:** Baseline clinical and obstetric characteristics according to disease activity trajectories during pregnancy.

Characteristics	Persistent Low Activity (*n* = 155)	Early Flare with Control (*n* = 43)	Uncontrolled Disease Activity (*n* = 47)	*p* Value
Maternal age (years)	30.3 ± 3.7	30.1 ± 3.9	31.1 ± 3.7	0.351
History of lupus nephritis, *n* (%)	36 (23.2%)	13 (30.2%)	27 (57.4%)	0.001 **
History of NP-SLE, *n* (%)	1 (0.6%)	0 (0.0%)	1(2.1%)	0.595 ^Δ^
aPL positivity, *n* (%)	52 (33.5%)	22 (51.2%)	29 (61.7%)	0.001 **
Hydroxychloroquine use, *n* (%)	141 (91.0%)	39 (90.7%)	41 (87.2%)	0.75
Steroid dose >10 mg/day, *n* (%)	19 (12.3%)	38 (88.4%)	38 (80.9%)	0.001 **
Parity (nulliparous), *n* (%)	73 (47.1%)	21 (48.8%)	18 (38.3%)	0.514
History of prior adverse pregnancy outcomes, *n* (%)	20 (12.9%)	6 (14.0%)	7(14.9%)	0.936

** *p* < 0.01. Data are presented as mean ± SD or n (%), as appropriate. aPL, antiphospholipid antibodies, aPL positivity indicates any positive aPL test (LA/aCL/anti-β2GPI); ^Δ^ Due to sparse counts, *p* values were obtained using a Monte Carlo exact test (two-sided).

**Table 2:** Pregnancy outcomes according to disease activity trajectories.

Outcomes	Persistent Low Activity (*n* = 155)	Early Flare with Control (*n* = 43)	Uncontrolled Disease Activity (*n* = 47)	*p* Value
Clinically actionable pregnancy complications, *n* (%)	6 (3.9%)	5 (11.6%)	30 (63.8%)	0.001 **
Spontaneous preterm birth, *n* (%)	29 (18.7%)	5 (11.6%)	9 (19.1%)	0.53

** *p* < 0.01. Clinically actionable pregnancy complications were defined as medically indicated preterm birth (<37 weeks), severe preeclampsia requiring delivery, or maternal organ dysfunction leading to pregnancy termination. Components were not mutually exclusive.

**Table 3:** Multivariable logistic regression analysis of clinically actionable pregnancy complications.

Variables	Adjusted OR	95% CI	*p* Value
Disease activity trajectory			
Early flare with control vs. persistent low	1.03	0.24–4.49	0.97
Uncontrolled disease activity vs. persistent low	13.45	4.01–45.08	<0.001 **
Maternal age (per year)	1.05	0.92–1.20	0.45
History of lupus nephritis	1.83	1.15–2.93	0.01 *
aPL positivity	1.91	0.74–4.95	0.18
Hydroxychloroquine use	0.82	0.19–3.54	0.79
Steroid dose > 10 mg/day	4.42	1.33–14.76	0.02 *

* *p* < 0.05, ** *p* < 0.01. Persistent low disease activity was used as the reference category. Models were adjusted for maternal age, history of lupus nephritis, antiphospholipid antibody status, hydroxychloroquine use, and steroid dose.

## Data Availability

The original contributions presented in this study are included in the article. Further inquiries can be directed to the corresponding authors.
